# Temperature- and pH-Responsive Poly(NIPAM-*co*-HEMA-*co*-AAm) Nanogel as a Smart Vehicle for Doxorubicin Delivery; Combating Colorectal Cancer

**DOI:** 10.3390/gels11040227

**Published:** 2025-03-22

**Authors:** Soheila Ghasemi, Mehdi Najafi, Mohammad Doroudian, Banafsheh Rastegari, Abbas Behzad-Behbahani, Hadis Soltanimehr, Fatemeh Farjadian

**Affiliations:** 1 Department of Chemistry, College of Sciences, Shiraz University, Shiraz 7194684795, Iran; ghasemis@shirazu.ac.ir (S.G.); mehdi.nj1372@gmail.com (M.N.); soltanimehr.hadis@gmail.com (H.S.); 2 Department of Cell and Molecular Sciences, Faculty of Biological Sciences, Kharazmi University, Tehran 1571914911, Iran; 3 Diagnostic Laboratory Sciences and Technology Research Center, School of Paramedical Sciences, Shiraz University of Medical Sciences, Shiraz 7143918596, Iran; brastegari@sums.ac.ir (B.R.); behzadba@sums.ac.ir (A.B.-B.); 4 Pharmaceutical Sciences Research Center, School of Pharmacy, Shiraz University of Medical Sciences, Shiraz 7146864685, Iran

**Keywords:** hydrogel, smart vehicle, RAFT polymerization, PNIPAM, doxorubicin, drug delivery

## Abstract

In this project, a new class of temperature- and pH-sensitive hydrogel consisting of *N*-isopropyl acrylamide (NIPAM), hydroxyethyl methacrylate (HEMA), and acrylamide (AAm) was prepared via a controlled route through the reversible addition–fragmentation chain-transfer (RAFT) polymerization process. Poly(ethyleneglycol) dimethacrylate (PEG-DMA) was used as a long-chain hydrophilic and biocompatible crosslinking agent. The hydrogel structure was confirmed by different characteristic techniques such as ^1^H NMR, FT-IR, and SEC, and the morphology and particle diameters were checked via the scanning electron microscopy (SEM) and dynamic light scattering (DLS) methods. Afterward, the as-prepared hydrogel, poly(NIPAM-*co*-HEMA-*co*-AAm), was loaded with doxorubicin (DOX) to be used as a temperature- and pH-triggered delivery carrier. The prepared system released DOX slowly at 37 °C and neutral pH, but increased DOX release significantly at 42 °C and acidic pH. The anti-cancer efficiencies of free DOX, hydrogel, and the DOX–hydrogel conjugate were tested in vitro using human colorectal adenocarcinoma HT-29 cell lines. Cytotoxicity evaluation of free DOX compared with the DOX–hydrogel conjugate revealed that more cancer cells were killed with increasing concentration. Moreover, the DOX-mediated apoptosis and ROS levels showed the beneficial effects of poly(NIPAM-*co*-HEMA-*co*-AAm) hydrogel for cancer drug delivery. Generally, the results suggest that this system can be a potential candidate for designing drug delivery systems.

## 1. Introduction

The application of nanotechnology as an emerging field in the biomedical sciences and health care systems has come to be known as “nanomedicine”. This field of science has played a pivotal role in finding critical solutions to significant problems in medicine, including therapy, diagnosis, and tissue engineering [1,2]. To date, more than 100 nano-pharmaceuticals have been accepted by the US Food and Drug Administration, and the numbers are on the rise every year [3].

Cancer, a source of mortality worldwide, caused 10 million deaths in 2020. Common categories of cancer are breast, cervical, and ovarian, mostly in females; prostate in males; bladder; colon; lung; and skin [4]. Colorectal cancer (CRC), also known as colon cancer, is a harmful disease that is widely prevalent and common in developing countries [5,6]. Doxorubicin (DOX) has been extensively studied for its efficacy in cancer therapy and is primarily included in the chemotherapeutic regimen of various cancers [7]. Despite its many benefits, innovation in its release is required due to its adverse side effects on healthy organs and lack of efficacy in the tumor area [8].

A unique approach in nanomedicine is drug delivery, which refers to the safe transport of a drug to a specific part of the body. This approach has made significant advances in cancer treatment, allowing anti-cancer drugs to be delivered directly to tumor sites without harming healthy organs [9,10]. Engineered biosafe nanoparticles of various origins, such as polymers, silica, graphene, iron oxides, gold, and quantum dots, have been developed and used for drug release in cancer treatment [11]. Polymeric nanoparticles in the form of micelles, hydrogels, molecular imprints, and dendrimers have been commonly used in the design of carriers for drug and gene liberation [12,13]. Hydrogels, as crosslinked 3-D polymeric networks with high capacity to adsorb water and therapeutic agents, have attracted the attention of researchers [14]. On the other hand, smart nanogels such as nanosized colloidal hydrogels have been paved the way for numerous applications in drug, gene, and vaccine delivery [15].

Smart stimuli-responsive hydrogels are recognized as state-of-the-art platforms for biomedical approaches, including drug delivery systems (DDSs). The ability of their structure to respond to physical and chemical stimuli is a key parameter in their utility. A fundamental structure in DDS design is the dual temperature- and pH-responsive hydrogel. Temperature-responsive carriers have an adjusted lower critical solution temperature (LCST) such that they have an expanded structure at average body temperature (37 °C) and can shrink and release drugs above this temperature, which is sensible at tumor sites or can be imposed upon solid tumors by an external heat source [16]. Poly(*N*-isopropylacrylamide) (PNIPAM) has an aqueous LCST of 32 °C and is a temperature-sensitive polymer. PNIPAM’s LCST can be tuned to be around the body’s average temperature by adding hydrophilic segments in the structure [17]. Acidic pH is another critical parameter in the environment of cancerous tumors, which differ from healthy organs that maintain a physiological pH. pH-responsive carriers, which contain acidic or basic groups, can deliver cargo while sensing pH changes in cancer cells [18].

The synthesis of nanohydrogels was made possible by the revolution in chain control through the controlled radical polymerization (CRP) technique [19]. The most recent and rapidly developing method in CRP is the RAFT method. In this process, a reagent with a thiocarbonylthio moiety is used as a RAFT agent [20]. The compatibility of RAFT with a wide diversity of monomers, including acrylates, meth(acrylates), styrene, and their derivatives, allows the creation of well-defined macromolecular libraries through the coupling of different monomeric components [21].

Hydrogels are being extensively investigated as DOX delivery systems. A chitosan-albumin-based nanogel was synthesized and assessed for DOX delivery in skin cancer. This bio-formulation stabilized the DOX against degradation under photo irradiation and enhanced the anti-neoplastic effect on the skin cancer cells in a pH-sensitive manner [22]. An implantable DDS based on methacrylate glycol chitosan was applied for DNA/DOX release in postsurgical breast cancer for both treatment and immunotherapy purposes. In vitro and in vivo results have revealed that this platform could suppress tumor recurrence, inhibit metastasis, and improve treatment outcomes [23]. An injectable smart hydrogel of alginate and poly(N-acroyl glycinamide) entrapped with DOX was prepared. The hydrogel showed thermal and near-infrared (NIR) responsiveness. After NIR irradiation, high DOX release was observed at 37 °C [24]. Several reports on the preparation of DOX DDSs based on synthetic hydrogels made with the RAFT protocol were reported. Lysin-modified poly(N-vinyl caprolactam) hydrogel crosslinked with poly(ethyleneglycol dimethacrylate) was applied for thermal- and pH-responsive DOX delivery and tested in vivo on breast cancer cells [25]. In another study, pH-responsive hydrogel based on poly(hydroxyethylmethacrylate-*co*-*N*,*N*′-dimethylaminoethyl methacrylate) was utilized for the same purpose [26]. A hybrid core–shell structure made of a silica core and pH-responsive polymethacrylic acid hydrogel was reported to be a sustained DDS [27].

In continuation of our previous reports on RAFT polymerization and smart hydrogels synthesis and their application as DDSs [28,29,30,31], and due to the unique properties of hydrogels [32], a temperature- and pH-sensitive nanohydrogel poly(*N*-isopropylacrylamide-*co*-hydroxyethylmethacrylate-*co*-acrylamide) (poly(NIPAM-*co*-HEMA-*co*-AAm)) is herein introduced as a smart vehicle for DOX delivery.

## 2. Results and Discussion

### 2.1. Synthesis and Characterization of Poly(NIPAM-co-HEMA-co-AAm) Hydrogel *(**I**)*

The RAFT polymerization method is attractive for fabricating novel materials with different characteristics. This is a kind of polymerization in which monomers react in a controlled process in the presence of a dithiocarbonyl compound as a CTA. A RAFT polymerization system was employed to fabricate polymeric carriers with predetermined chain length and dispersity.

**Scheme 1:**
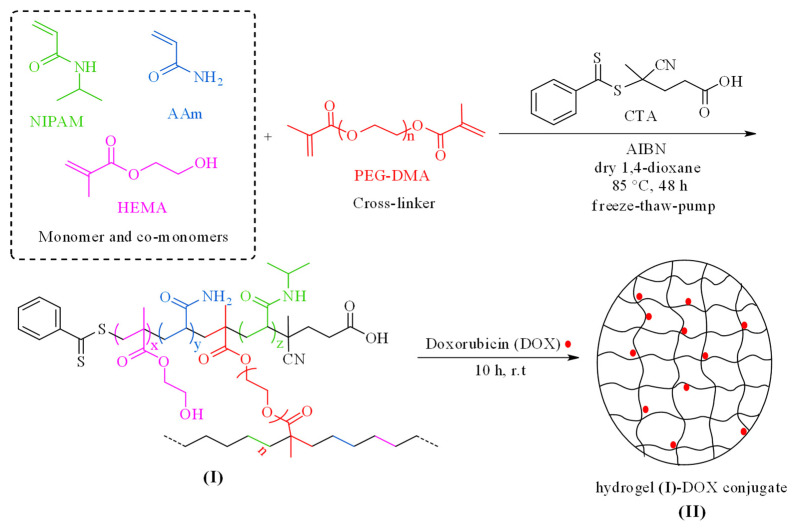
Schematic depiction for the preparation of poly(NIPAM-*co*-HEMA-*co*-AAm) hydrogel (**I**) and the corresponding hydrogel–DOX conjugate (**II**).

Herein, the NIPAM monomer was recruited for the synthesis of a smart temperature-responsive nanogel (compound **I**). To improve the hydrophilicity and proper swelling of the system in water and to adjust the LCST of the synthesized nanocarrier in the temperature range of 37 °C, HEMA and AAm *co*-monomers were utilized. HEMA can absorb water-solubilized pharmacological agents up to 50% of its weight and can passively diffuse into surrounding media upon in vitro application. Other advantages of HEMA like nontoxicity, biodegradability, biocompatibility, high specific surface area, resistance to acidic media, and its suitable structure as a drug carrier make it a good candidate as a *co*-monomer in the design of this smart nanovehicle. A copolymer system composed of PNIPAM and PHEMA could be engineered for synergistic drug delivery. In such systems, PNIPAM may contribute to temperature-sensitive drug release, while PHEMA could add pH sensitivity. The acidic conditions can reduce the interactions between the drug and the polymer, promoting drug release [33,34,35]. Additionally, PAAm and PHEMA enhance the permeability of water and oxygen within this system, which is crucial for swelling behavior, drug release, cell interactions, and effective tumor treatment. PEG-DMA is a long-chain, hydrophilic, biocompatible, and environmentally friendly crosslinking agent used to synthesize this nanogel. Furthermore, the CTA that was applied in this reaction was regarded as a suitable reagent for the polymerization of acrylate and methacrylate monomers in the presence of AIBN as an initiator in the RAFT process. Poly(NIPAM-*co*-HEMA-*co*-AAM) hydrogel-loaded DOX (**II**), as an antitumor agent, was produced by treating compound (**I**) with DOX (Scheme 1). The resultant product was purified by dialysis and characterized by various techniques.

**Figure 1:**
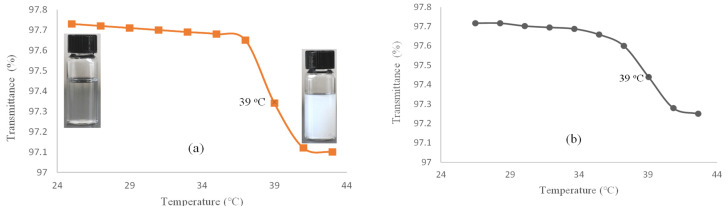
Transmittance (%) versus temperature (°C) for (**a**) hydrogel (**I**) and (**b**) hydrogel–DOX conjugate (**II**) solution.

For evaluating the LCST, the percent of transmittance of the hydrogel (**I**) solution in deionized (DI) water is measured versus temperature by using a UV–Vis spectrophotometer (Figure 1a). This was nearly constant in the temperature range of 25–37 °C. Thereafter, the solution became turbid at 38 °C and continued to 41 °C. Similarly, the hydrogel–DOX conjugate (**II**) showed a phase transition at 38 °C, which continued up to 41 °C (Figure 1b). The hydrogel phase transition occurs due to the simultaneous presence of amide and isopropyl groups as hydrophilic and hydrophobic components in the NIPAM structure. Above the LCST, the solution becomes turbid as the hydrogel structure collapses due to hydrophobic interactions.

**Figure 2:**
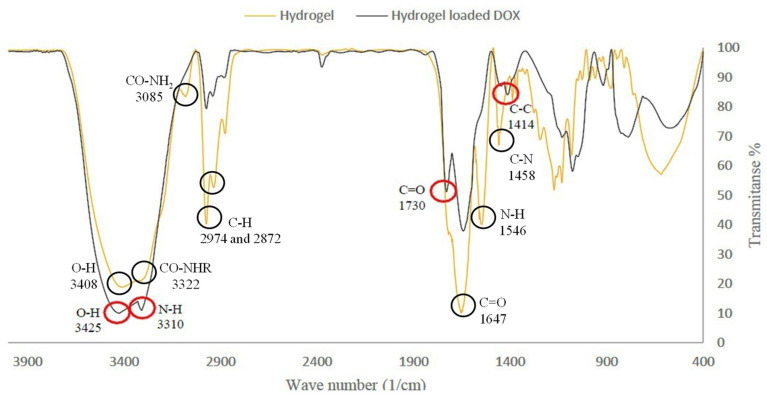
FT-IR spectrum of hydrogel (**I**) and hydrogel loaded DOX (**II**).

The FT-IR spectrum of the hydrogel (**I**) showed the characteristic absorption bands of the hydroxyl group (-OH) at 3408, secondary amide group (-CO-NHR) at 3322, primary amide group (-CO-NH_2_) at 3385, (-NH) bending at 1546, carbonyl group (-C=O) at 1647, and (-C-N) stretching at 1458 cm^−1^. The signal at 2974 cm^−1^ was due to the stretching absorption of aliphatic groups (Figure 2). The FT-IR spectrum of the hydrogel–DOX conjugate (**II**) exhibited the characteristic absorption of the -OH stretching of HEMA at 3425, and the amide group (-N-H) at 3310 cm^−1^. DOX peaks included the carbonyl functional group (-C=O) at 1730 and the C=C stretching of the aromatic ring at 1414 cm^−1^ (Figure 2).

The presence of RAFT agent in the structure of the hydrogel (**I**) and the average degree of polymerization (DP) and molecular weight of compound (**I**) were obtained from ^1^H-NMR spectroscopy. The ^1^H-NMR spectrum of poly(NIPAM-*co*-HEMA-*co*-AAM) hydrogel (**I**) in D_2_O is represented in the Supporting Information (Supplementary Materials, Figure S1). Aromatic hydrogens of 4-cyano-4-(phenylcarbonothioylthio)pentanoic acid appear at 7.38, 7.55, and 7.87 ppm, confirming the presence of RAFT agent in the hydrogel structure. The average DP of PNIPAM was calculated by comparing the relative intensities of signals at 7.38–7.87 ppm related to the aromatic regions of RAFT agent with the signal of isopropyl proton of the NIPAM repeating unit at 3.64 ppm. The DP_n_, _NMR_ of NIPAM from this measurement is obtained as 62.85. Similarly, the average DP of HEMA and AAm repeating units were obtained by comparison of relative intensities of signals at 7.38–7.87 ppm related to the aromatic regions of RAFT agent with the signal at 3.58 and 2.1 ppm due to HEMA and AAm repeating units, respectively. From these assessments, the DP_n_, _NMR_ of HEMA and AAm are calculated 16.23 and 26.66, respectively. There is a good conformity between the results of number-average molecular weights obtained from ^1^H-NMR spectroscopy evaluation and theoretical calculations. The results are demonstrated in Table S1 (see Supplementary Materials). These results suggest that the 4-cyano-4-(phenylcarbonothioylthio) pentanoic acid is an efficient RAFT agent for NIPAM, HEMA, and AAm CRP.

**Figure 3:**
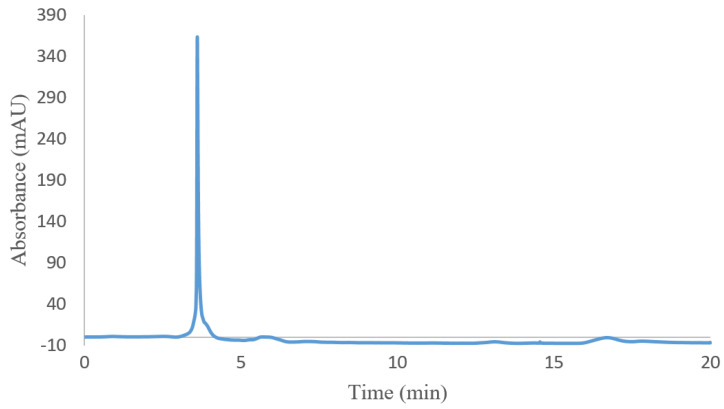
SEC chromatogram of poly(NIPAM-*co*-HEMA-*co*-AAm) hydrogel (**I**).

For evaluating the molecular weight of hydrogel (**I**), size-exclusion chromatography (SEC) was carried out. The SEC chromatogram of hydrogel (**I**) is shown in Figure 3. The retention time of hydrogel (**I**) is 3.6 min. Based on a comparison with the calibration curve of standard samples, the hydrogel molecular weight is around 12.520 KD.

The DLS method was also used to determine the hydrodynamic size of hydrogel (**I**). As shown in Figure S2, the DLS test revealed the estimated hydrodynamic diameter of hydrogel (**I**) before, after, and at its LCST. At an ambient temperature of 25 °C (lower than LCST), the largest aggregated hydrogel (**I**) had an average size of 111 nm, whereas at around its LCST (39 °C) and higher than LCST (55 °C), aggregates with sizes of approximately 307 and 858 nm were found (Supplementary Materials, Figure S2).

**Figure 4:**
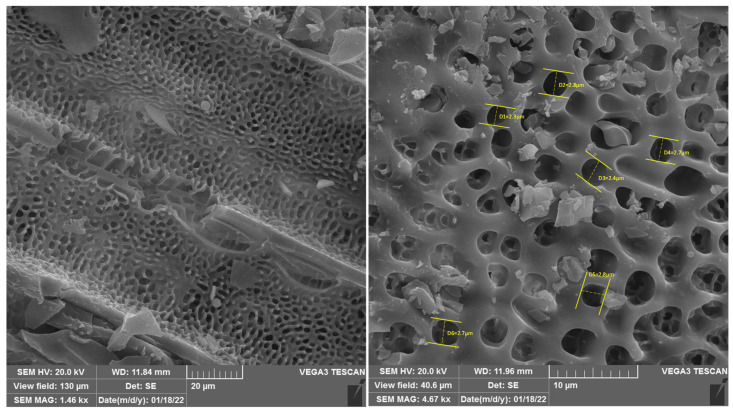
SEM images of hydrogel (**I**).

Scanning electron microscopy (SEM) was applied to envisage the probable macroscopic structures and morphology of hydrogel (**I**) (Figure 4). The SEM images displayed microstructures with a uniform porous structure. As illustrated in the SEM micrographs, the pore size detected in hydrogel (**I**) was about 2.3–2.8 μm.

### 2.2. Adsorption Study of DOX

**Figure 5:**
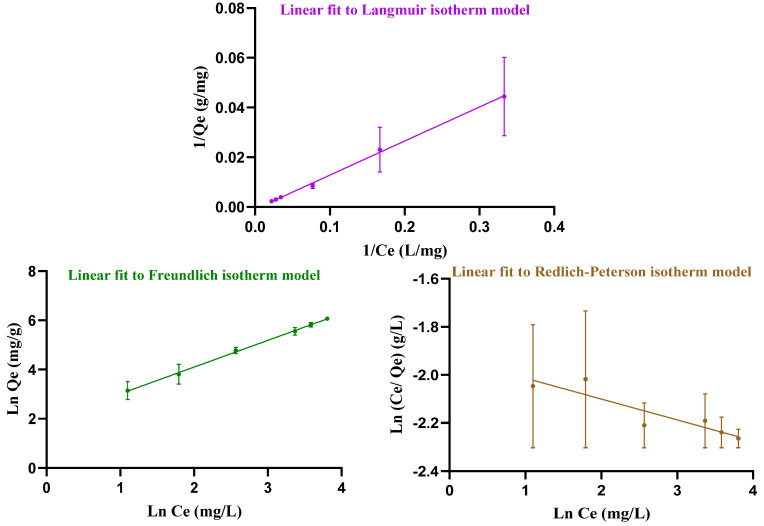
Linear Langmuir, Freundlich, and Redlich–Peterson isotherm models, fitted to the drug loading data from the experiment.

Adsorption occurs when soluble substances, called adsorbates, bind to a solid adsorbent. To investigate how DOX is loaded, the data were analyzed using three adsorption models: Langmuir, Freundlich and Redlich–Peterson (Figure 5). These isotherms describe the relationship between the amount of adsorbate (DOX) and the adsorbent (hydrogel). The Langmuir isotherm assumes, firstly, that adsorptive sites have equivalent energies. Secondly, there is a fixed number of adsorptive sites that results in monolayer adsorption in homogeneous systems. Thirdly, only one adsorbate species can be housed onto a single site [36]. In contrast, the Freundlich isotherm is related to the multisite adsorption on heterogeneous surfaces. The Redlich–Peterson isotherm describes the adsorption of organic compounds or gases on porous adsorbents, incorporating features of both the Langmuir and Freundlich isotherms. It can be used in homogeneous as well as heterogeneous systems [36]. Table 1 reviews the results for the Langmuir, Freundlich, and Redlich–Peterson constants attained from Equations (5)–(7). In this experiment, the Freundlich isotherm model, with its higher R^2^ value, provides a better fit for the loading data than the other isotherms and indicates multisite adsorption.

### 2.3. DOX Drug-Release Behavior Investigation

Eco-friendly smart hydrogels have great potential in various applications, including smart drug delivery vehicles, due to their responsiveness to external stimuli such as temperature, pH, light, magnetic and electric fields, etc. [37]. Elevated temperatures and low pH are environmental variables detected in the body. Accordingly, pH- and temperature-sensitive hydrogels can be used in drug delivery systems to provide site-specific and controlled release of biologically active agents [38].

**Figure 6:**
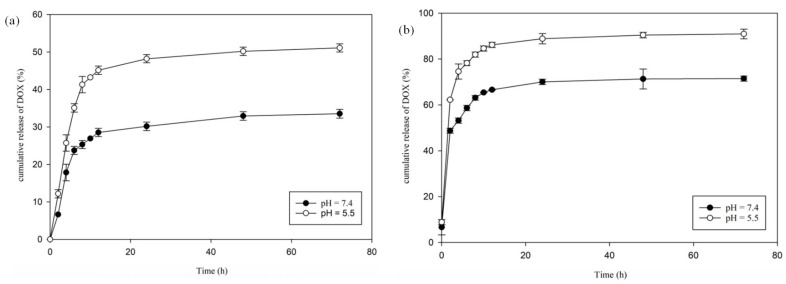
DOX releases at different pH values at (**a**) 37 °C (below LCST) and (**b**) 42 °C (above LCST) at different time intervals within 72 h.

To gauge the temperature- and pH-sensitive feature of hydrogel–DOX conjugate (**II**), the delivery behavior of the as-synthesized hydrogel was investigated. The function of DOX release from hydrogel was explored at two pH values of 7.4 (pH of typical tissue environment) and 5.5 (pH of tumor tissue environment) and two different temperatures of 37 °C (≥the LCST) and 42 °C (≤the LCST) at various time intervals within 3 days (72 h), and the release profiles were represented. As depicted in Figure 6, the behavior of DOX delivery from the hydrogel–DOX (**II**) revealed apparent variation with changes in temperature and pH. At 37 °C (lower than LCST), with pH 7.4 and 5.5 (pH of normal and cancer tissues), 33 and 51% of DOX were released from the hydrogel after 72 h, respectively. More DOX delivery was observed in pH 5.5 in comparison with pH 7.4 due to the DOX release from the carrier being greater in acidic media. When the temperature increased to 42 °C, the total amount of DOX released in pH 7.4 and 5.5 from the support after 72 h was about 71 and 91%, respectively (Figure 6). The release of DOX from hydrogel–DOX is nearly complete at 42 °C and pH 5.5 (91%), but it is small-scale at 37 °C and pH 7.4 (33%).

The collapse and swelling of hydrogel (**II**) in an aqueous environment may explain the different DOX release behavior at two temperature values (below and above LCST). The increasing hydrophobic character of the PNIPAM chain with increasing temperature induces a low solubility of the hydrogel in water, destabilizing the hydrogel. Consequently, the LCST of the hydrogel may affect the drug release pattern from the hydrogel at its transition temperature.

In addition, to study the sustained release mechanism and kinetics of the drug carrier, the Higuchi Equation (1) and Korsmeyer–Peppas Equation (2) models were used to analyze and predict DOX release in DDS. Their expression of the formula is as follows:

Q_t_/Q_∞_ = k_H_t^1/2^ (1)

Q_t_/Q_∞_ = k_KP_t^*n*^ (2)

where Q is the cumulative amount of drug liberated at time t, Q_∞_ is the maximum cumulative release, and k_KP_ and k_H_ are the Korsmeyer–Peppas and Higuchi rate constants, respectively.

**Figure 7:**
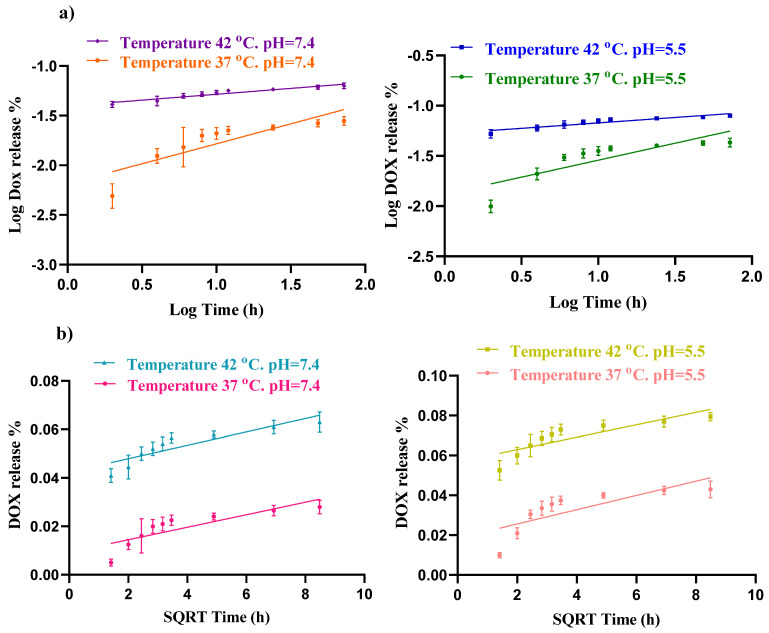
Fitting drug release data of hydrogel loaded DOX to (**a**) Korsmeyer–Peppas and (**b**) Higuchi models at different pH (7.4 and 5.5) and temperatures (37 and 42 °C).

The Higuchi kinetic model describes the release of drugs from a solid matrix system and explains how the drug diffuses through the matrix over time. It is one of the earliest and simplest models for controlled drug release and is particularly useful for systems where drug release is governed by Fickian diffusion. The diffusion exponent (*n*) in the Korsmeyer–Peppas model also indicates the mechanism of drug release. Fickian diffusion occurs when *n* is between 0 and 0.45, whereas anomalous diffusion occurs when *n* ranges from 0.45 to 0.89. Fickian diffusion follows Fick’s laws, whereas non-Fickian diffusion does not [39]. In this experiment, the Korsmeyer–Peppas model, with its higher R^2^ value, provides a better fit for the DOX release data than the Higuchi model, suggesting that the primary mechanism of DOX delivery from this carrier is Fickian diffusion (Figure 7).

### 2.4. In Vitro Cellular Studies

**Figure 8:**
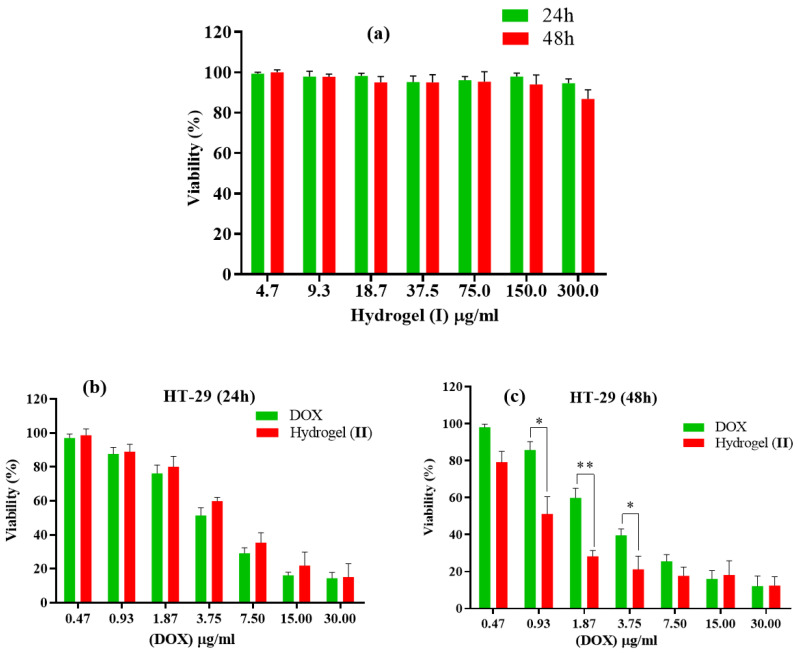
Cell toxicity against HT-29 of (**a**) hydrogel (**I**) for 24 and 48 h duration; (**b**,**c**) free DOX and hydrogel–DOX conjugate (**II**) within (**b**) 24 h and (**c**) 48 h. The statistical levels of significancy are presented as follows: * *p* < 0.05, ** *p* < 0.01.

The anti-cancer efficacy of free DOX, hydrogel (**I**), and hydrogel–DOX conjugate (**II**) were tested in vitro. HT-29 cells were treated with the three ingredients mentioned in each set. After completing the MTT test, the viability percentage of all fractions was evaluated during two distinct time intervals, 24 h and 48 h, and at seven different doses of DOX ranging from 0.47 to 30.00 µg/mL (Figure 8). In the 24 h investigations, when free DOX was compared to an equivalent quantity in conjugated form (**II**), the latter showed lower cytotoxicity compared to the free DOX. This may be due to the controlled release of the DOX from hydrogel. After 48 h of testing, there were signs of better viability with different concentrations, but percentage values dropped significantly. According to the IC_50_ values of each condition, the IC_50_ value of free DOX was estimated to be 4.91 ± 1.94 µg/mL and 3.72 ± 1.48 µg/mL within 24 and 48 h after treatment, respectively. While the free DOX treatment showed a closed IC_50_ value, hydrogel–DOX conjugate (**II**) showed a remarkable improvement of the IC_50_ values from 6.15 ± 2.36 µg/mL to 1.29 ± 0.87 µg/mL after 24 and 48 h treatment, respectively.

### 2.5. Apoptosis Induced Cell Death

**Figure 9:**
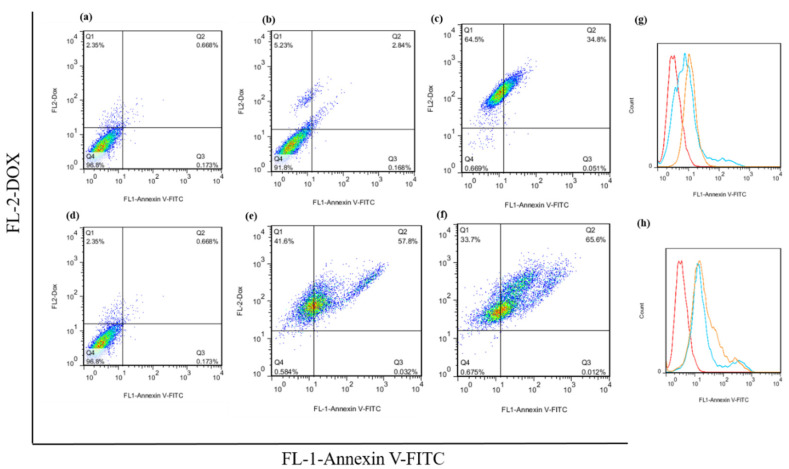
The apoptotic rate of the HT-29 untreated cells, cells after treatment with (**a**) untreated cells, (**b**) free 1×IC_50_ and (**c**) 2×IC_50_ of DOX, (**d**) untreated cells, and (**e**) 1×IC_50_ and (**f**) 2×IC_50_ of hydrogel (**II**). The histogram plot of the green fluorescence emission of the Annexin V-FITC after treatment with (**g**) free DOX and (**h**) hydrogel (**II**) are presented.

The flow cytometry analysis of the apoptosis assay of the free DOX and hydrogel–DOX conjugate (**II**) against the HT-29 cell line are presented in Figure 9. The dot plot analysis shows a significant alteration in the apoptosis rate between the control group and the treatment groups that received 2×IC_50_ concentrations of free DOX (*p* = 0.021). Moreover, using hydrogel–DOX conjugate (**II**), the differences in apoptosis rate were dramatically elevated, which was statistically significant in both 1×IC_50_ and 2×IC_50_ values (*p* = 0.0008, and *p* < 0.0001). These results suggest that the hydrogel–DOX conjugate (**II**) was more effective than free DOX in inducing programmed cell death through apoptosis in cancer cells.

### 2.6. Cellular Uptake

**Figure 10:**
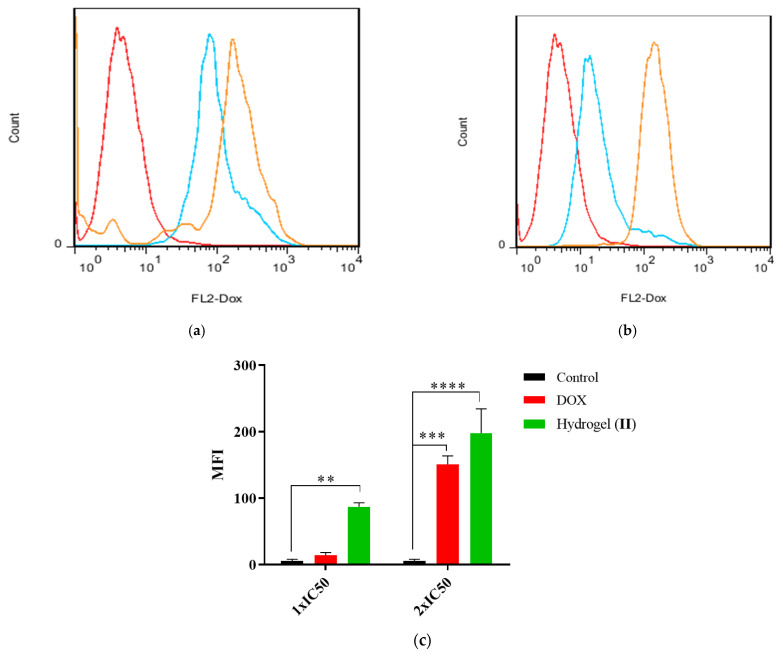
The histogram plots of HT-29 cells after treatment with (**a**) free DOX and (**b**) hydrogel–DOX conjugate (**II**) at 1×IC_50_ and 2×IC_50_ concentrations within 6 h; (**c**) the comparative plots of MFI after three independent experiments. The statistical levels of significancy are presented as follows: ** *p* < 0.01, *** *p* < 0.001; and **** *p* < 0.0001.

The cellular uptake of hydrogel–DOX conjugate (**II**) against HT-29 cell lines is presented in Figure 10. The greater fluorescence intensity in 1×IC_50_ concentrations in the compound (**II**) population suggests improved drug incorporation into the target cells. As shown in Figure 10a, the fluorescence emission of the HT-29 cells was slightly shifted after treatment with free DOX, while after treatment with 2×IC_50_, the shift was significantly elevated compared to control (*p* = 0.006). The treatment of HT-29 cells with hydrogel–DOX conjugate (**II**) also showed a different pattern even at 1×IC_50_ value of the DOX. The MFI indicated a significant accumulation of DOX in HT-29 cells in both 1×IC_50_ and 2×IC_50_ compared to the control cell population (*p* = 0.0007 and *p* < 0.0001).

### 2.7. ROS Production

**Figure 11:**
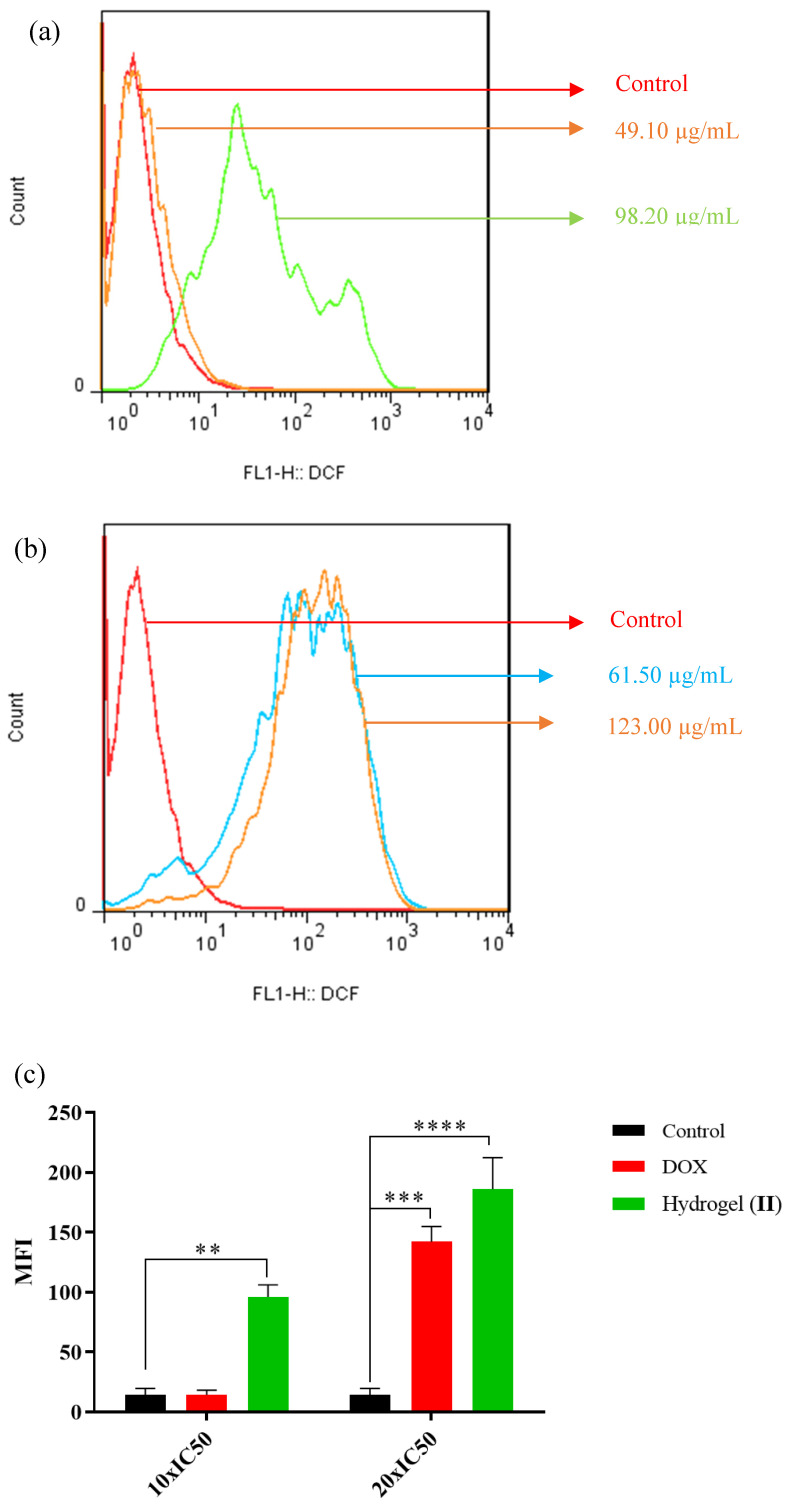
The relative spectra of green fluorescent intensity of DCF followed by incubation with (**a**) DOX and (**b**) hydrogel–DOX conjugate (**II**) for 4 h, (**c**) the comparative plots of MFI have been presented after three independent experiments.The statistical levels of significancy are presented as follows: ** *p* < 0.01, *** *p* < 0.001; and **** *p* < 0.0001.

Figure 11 shows the green fluorescence emission resulting from the intracellular ROS generation of HT-29 mitochondria. Cells were exposed to free DOX (49.1, 98.2 µg/mL) and hydrogel–DOX conjugate (**II**) (61.5 and 123.0 µM) for 4 h. As illustrated in Figure 11a, the fluorescence emission of the HT-29 cells was slightly shifted after treatment with a lower dosage of the free DOX, while after treatment with a high concentration, the shift was significantly elevated compared to control (*p* = 0.008). The treatment of HT-29 cells with hydrogel–DOX (**II**) also showed different patterns even at lower concentrations of the DOX. The MFI indicated significant ROS generation in HT-29 cells in low and high concentrations compared to the control cell population (*p* = 0.0009 and *p* < 0.0001).

## 3. Conclusions 

Herein, a temperature- and pH-sensitive poly(NIPAM-*co*-HEMA-*co*-AAm) hydrogel was prepared via RAFT polymerization to serve as a drug carrier for DOX. In this smart nanogel, NIPAM was used as a thermo-responsive monomer, while HEMA and AAm acted as hydrophilic *co*-monomers to effectively adjust the LCST of the system. Under physiological conditions, the polymer-loaded DOX was hydrophilic, but underwent a phase transition when heated above its LCST of 39 °C. This property enabled selective drug uptake by tumor cells, exploiting the temperature difference between normal and cancerous tissues. The release pattern of DOX showed over 90% drug release at pH 5.5 and 42 °C. The hydrogel demonstrated high toxicity comparable to free DOX in HT-29 cells over 24 and 48 h. The carrier’s temperature-responsiveness played a key role in controlling the drug release at 40 °C. Additionally, the hydrogel–DOX conjugate (**II**) outperformed free DOX in inducing apoptosis, enhancing drug uptake, and increasing ROS production in HT-29 cells, highlighting its potential for targeted cancer therapy. Future improvements could focus on optimizing drug loading, release profiles, biocompatibility, functionalization, toxicity studies, combination therapies, clinical translation, personalized medicine, and integration with imaging techniques, paving the way for more effective, targeted, and minimally invasive cancer treatments.

Future research related to drug-loaded hydrogels will be multidisciplinary, encompassing materials science, drug formulation, biotechnology, and clinical application. By optimizing hydrogel formulations, improving drug loading capacities, and conducting thorough in vivo studies, these advanced drug delivery systems hold the potential to revolutionize how drugs are delivered, offering more precise, efficient, and safer therapeutic options for a variety of diseases.

## 4. Materials and Method

### 4.1. Materials and Equipment

Azobisisobutyronitrile (AIBN, initiator, Merck 98%) was purified by recrystallization from methanol. Acrylamide (AAm, *co*-monomer, Fluka 97%) and *N*-isopropyl acrylamide (NIPAM, monomer, Acros 99%) were recrystallized from chloroform and *n*-hexane, respectively. The 2-Hydroxyethyl methacrylate (HEMA, *co*-monomer), PEG dimethacrylate (PEG-DMA, crosslinking agent, MW = 550), and 4-cyano-4-(phenylcarbonothioylthio)pentanoic acid as RAFT agent was obtained from Sigma-Aldrich (Burlington, MA, USA). The RAFT agent was dried under vacuum before use, and HEMA and PEG-DMA were purified by passing through a short silica column and kept at −10 °C under an N_2_ atmosphere. The 1,4-Dioxane anhydrous (Sigma, 99.8%) and diethyl ether (Sigma, ≥99.0%) were pre-dried with calcium hydride and then heated at reflux for several hours in the presence of Na wire and benzophenone (1% and 0.2% *w*/*v*, respectively) until the solvent turned deep blue. Doxorubicin hydrochloride (DOX) was purchased from Pharmacia and used as received. DI water is employed throughout this study whenever necessary. Phosphate-buffered saline (PBS) is a buffer solution applied in biological research to produce adjusted pH solutions. In this study, a Gibco^™^ 18912014 PBS tablet (free from magnesium, calcium, and phenol red) was dissolved in 500 mL of distilled water to prepare a buffer solution with a pH of 7.45. The dialysis tubing used is a trial kit Spectra/Por^®^ Biotech CE, 12 kDa MWCO, 40 or 42 mm flat-width, and hydrogel dialysis was performed for around five hours. All other employed solvents and reagents were used as received with no further purification.

FT-IR data were acquired on a Shimadzu FTIR 8300 E spectrophotometer (Shimadzu Corporation, Kyoto, Japan) IR pellets were made ready by sample grinding with KBr using a pestle and mortar and subsequent pressing with a pellet press die set. The ^1^H-NMR spectra of compounds were recorded on a 400 MHz NMR spectrometer (Bruker Avance DPX model, Bruker Corporation, Billerica, MA, USA) in D_2_O solvent. Scanning electron microscopy (SEM) images were produced by the Tescan Vega3 (Tescan Orsay Holding, Brno, Czech Republic) apparatus at 20 kV. The SBC-12 small ion sputtering instrument (South Bay Technology, Inc., San Clemente, CA, USA) is mainly used to coat conductive gold film on freeze-dried samples for use in scanning electron microscopes using the physical vapor deposition (PVD) technique to obtain high-quality images. Dynamic light scattering (DLS) (Horiba SZ100-Z particle size analysis) was utilized to obtain the polymers’ hydrodynamic radius and particle distribution width. The DLS experiments were performed with aqueous samples (1–2 mg/mL) in 2 mL microtubes. An incubated shaker (JSH20 lur, 220 V, 12 A) was applied to admix samples and define drug releases at an appropriate temperature and during an intervention. For acquiring the UV data and absorbance measurements, a Cecil instruments CE 7200 UV/Visible spectrophotometer was used. For apoptosis/necrosis assays, cellular uptake, and ROS generation, Annexin V-FITC (eBioscience^™^, Invitrogen, Carlsbad, CA, USA) flow cytometry (BD Biosciences, San Jose, CA, USA) was applied. HT-29 cells were provided from Pasteur Inistitute of Iran (Tehran, Iran).

### 4.2. Preparation of Poly(NIPAM-co-HEMA-co-AAm) Hydrogel *(**I**)*

The nano hydrogel (**I**) was synthesized using NIPAM, HEMA, AAm, and PEG-DMA with a mole ratio of 60.5: 13.75: 25: 0.75. RAFT agent (16.7 mg, 0.0598 mmol), AIBN (initiator, 1.96 mg, 0.0119 mmol), PEG-DMA (cross-linker, 22.4 µL, 0.0447 mmol), NIPAM (monomer, 409.36 mg, 3.6175 mmol), HEMA (*co*-monomer, 100 µL, 0.8221 mmol), and AAm (*co*-monomer, 106.25 mg, 1.4948 mmol) (mole ratio of monomers: CTA: AIBN = 100:0.01:0.002) were dissolved in degassed and dried 1,4-dioxane (3 mL). The mixture was transferred to a Schlenk reaction tube containing a small magnetic stirrer and then purged with an inert gas like Ar for about 30 min before the reaction started. The content of the Schlenk tube was degassed seven times by freeze–pump–thaw (FPT) cycling entirely. By this means, the charged Schlenk tube was closed, and the contents were frozen in liquid nitrogen (LN_2_) and then put under a vacuum. Afterward, the flask was closed and the solvent was made warm to become liquid. Finally, all steps were repeated seven times. After degassing, the tube was sealed and heated in an oil bath at 85 °C for 48 h. This was followed by cooling, dilution with acetone, and quenching by precipitation in dry and absolute diethyl ether. Finally, to obtain hydrogel (**I**), the reaction mixture was centrifuged (2 × 4 min, 5000 rpm), washed thoroughly with diethyl ether, and dried under vacuum.

### 4.3. Adsorption Study of DOX with Hydrogel *(**I**)*

Poly(NIPAM-*co*-HEMA-*co*-AAm) hydrogel (**I**) (100 ppm) was loaded with varying concentrations of DOX (100, 150, 200, 300, and 500 ppm) by shaking in an incubator at 25 °C for a 24 h period. The values for entrapment efficiency (EE%) and loading capacity (LC%) were measured according to Equations (3) and (4) to determine the optimal hydrogel/drug ratio for further experiments. Additionally, DOX loading was analyzed using three adsorption isotherm models: Langmuir, Freundlich, and Redlich–Peterson according to Equations (5), (6), and (7), respectively.

EE% = (Mass of drug in hydrogel/Mass of total amount of drug initially used) × 100(3)

LC% = (Mass of drug in hydrogel/weight of hydrogel + drug) × 100(4)

1/q_e_ = 1/q_m_K_L_C_e_ + 1/q_m_ (5)

Log q_e_ = log K_F_ + 1/*n* log C_e_ (6)

ln (C_e_/q_e_) = alnC_e_ − lnK_RP_ (7)

where q represents the amount of DOX adsorbed (in mg) per gram of support at equilibrium, q_m_ indicates the maximum drug adsorption capacity, C_e_ is the equilibrium concentration of DOX, K_L_, K_F_, and K_RP_ are the constants for the Langmuir, Freundlich, and Redlich–Peterson models, respectively, and *n* is the experimental factor used in the adsorption study.

### 4.4. Preparation of Poly(NIPAM-co-HEMA-co-AAm) DOX-Loaded Hydrogel *(**II**)*

The poly(NIPAM-*co*-HEMA-*co*-AAm) hydrogel (**I**) (15 mg) was dissolved in DI water (4 mL), and DOX (30 mg) was then added while the mixture was stirred. The reaction was performed at ambient temperature for 24 h. The polymer–DOX conjugate was dialyzed in DI water for 5 h, and then the mixture was lyophilized in freeze-drier for 48 h. To define the DOX content, hydrogel (**I**) was dissolved in an aqueous acidic solution (pH 1.2) for 48 h, and the DOX content was quantified using UV–Vis spectroscopy based on the calibration curve method (Figure S3, Supplementary Materials).

### 4.5. DOX Drug Release Measurement

DOX/poly(NIPAM-*co*-HEMA-*co*-AAm) (4 mg, containing 367 µg of DOX) was suspended in 4 mL of PBS (pH = 5.5 or 7.4). Next, the suspension was conveyed into a dialysis membrane (12 KD MWCO, 40 or 42 mm flat-width). The dialysis bag was sunk in 50 mL PBS solution with pH = 7.4 or 5.5 and put in a shaker incubator at 37 °C and 42 °C. Then, 1 mL of an exterior solution of the dialysis membrane was withdrawn at different time intervals (i.e., 0, 0.5, 2, 4, 6, 8, 10, 12, 24, 48, 72 h), and the amount of liberated DOX was calculated by the UV–Vis technique at a wavelength of 485 nm. Following each evaluation, the external medium of the dialysis bag was removed and superseded with PBS, and each test was repeated 3 times. Total drug delivery data were determined as the amount of free DOX relative to the entire amount of DOX in hydrogel (**II**).

Cumulative percent of released DOX was measured as mentioned by the following equation:

(8) $$\mathrm{Cumulative}\;\mathrm{DOX}\;\mathrm{released}\;(\%)=\frac{V_{e}\sum_{1}^{n-1}C_{i}+V_{0}C_{n}}{m_{0}}\times 100\%$$

where V_0_ and V_e_ are the total volume of the release media (50 mL) and the delivery media that is removed each time (1 mL), C_i_ is the concentration of DOX in the delivering media, and m_0_ is the total mass of DOX loaded in the hydrogel (**II**) [40].

### 4.6. In Vitro Cellular Cytotoxicity Assessment

The MTT assay, which is based on the reduction of 3-(4,5-dimethyl thiazol-2-yl)-2,5-diphenyl tetrazolium bromide, was performed. Following this method, 100 µL of HT-29 cancer cells containing 1 × 10^4^ cells/well were cultured in 96-well plates containing fetal bovine serum (10%) and penicillin/streptomycin (1%) in RPMI 1640 for 16 h. Initially, the cell viability assay was performed to assess the cytotoxicity of free DOX through incubating cells with diverse concentrations of DOX ranging from 0.47 to 30 ppm (0.47, 0.93, 1.87, 3.75, 7.50, 15.00, 30.00 µg/mL) within 24 and 48 h. Furthermore, MTT tests were performed using an equal amount of compound (**II**), free DOX, and 4.7 to 300 ppm of compound (**I**) (4.7, 9.3, 18.7, 37.5, 75.0, 15.0, 30.0 µg/mL). After incubation, the medium was refreshed with 100 μL of 500 µg/mL of MTT solution in the culture medium. After 4 h, the medium was removed again, and the insoluble violate formazan was dissolved in 100% DMSO (100 μL/well) and finally evaluated with a microplate reader at λ = 570 nm and λ = 630 nm, related to the formazan and background absorption wavelengths, respectively. Three independent repeats were performed for each concentration at two periods of 24 and 48 h, and the cell viability was estimated by drug-treated cells relative to untreated control cells.

### 4.7. Apoptosis/Necrosis Assay

The total population of 2 × 10^5^ HT-29 cells was pre-cultured for 16 h. Cells were then exposed to 1×IC_50_ (4.91 µg/mL) and 2×IC_50_ (9.82 µg/mL) of DOX and 1×IC_50_ (6.15 µg/mL) and 2×IC_50_ (12.30 µg/mL) of compound (**II**) for 24 h, respectively. After that, fluorescein isothiocyanate (FITC)-tagged Annexin V was utilized to evaluate the apoptotic population cells using annexin V-FITC. Following the manufacturer’s protocol, cells were washed three times with 1000 μL of 1× binding buffer. Cells were then redissolved in 1× binding buffer (100 μL containing 5 μL annexin V-FITC) for 15 min. The apoptosis rates were then estimated by flow cytometry. The apoptosis percentage was considered as the apoptosis population rate (early apoptosis + late apoptosis) in the treated and control samples.

### 4.8. Cellular Uptake Procedure

The total number of 2 × 10^5^ HT-29 cells were pre-cultured for 16 h. After that, cells were treated with 1×IC_50_ and 1×IC_50_ (4.91 and 9.82 µg/mL) DOX and compound (**II**) (6.15 12.30 µg/mL) for 4 h. The red fluorescence intensity of the DOX was monitored using flow cytometry.

### 4.9. ROS Generation

Flow cytometric assessment of intracellular reactive oxygen species (ROS) production and mitochondrial membrane potential in HT-29 cells upon exposure to free radicals was performed. Free DOX (49.1 and 98.2 µg/mL) and compound (**II**) (61.5, 123.0 µM) were incubated for 4 h. The green fluorescence emission of treated cells was finally analyzed after treating cells with a DCFDA assay kit for 1 h using flow cytometry.

### 4.10. Statistical Analysis

Quantitative data were examined using two-way ANOVA. The levels of statistical significance were presented as *p*-values less than 0.05. The IC_50_ values of the MTT analysis and DCF fluorescence histogram were presented with the help of a mean ± S.D. obtained from three independent tests. An * indicates *p* < 0.05, ** *p* < 0.01, *** *p* < 0.001, and **** *p* < 0.0001 versus control.

## Figures and Tables

**Table 1:** Attained parameters from Freundlich, Langmuir and Redlich–Peterson models.

**Freundlich**	*n* K_f_ ((mg/g)/(mg/L)1/*n*) R^2^	17.95 1.17 0.98
**Langmuir**	q_m_ (mg/g) K_L_ (L/mg) R^2^	425.17 0.15 0.89
**Redlich–Peterson**	K_RP_ (L/g) a_RP_ R^2^	1.14 0.05 0.19

## Data Availability

The original contributions presented in this study are included in the article/Supplementary Material. Further inquiries can be directed to the corresponding authors.

## Supplementary Materials

The following supporting information can be downloaded at: https://www.mdpi.com/article/10.3390/gels11040227/s1, Figure S1: ^1^H-NMR spectrum of poly(NIPAM-*co*-HEMA-*co*-AAM) hydrogel (**I**) in D_2_O; Figure S2: DLS spectra showing the particle size distribution of hydrogel (**I**) at (a) 55 °C (T > LCST), (b) 39 °C (T = LCST), and (c) 25 °C (T < LCST); Figure S3: The calibration curve of the DOX. Table S1: RAFT polymerization of NIPAM, HEMA, and AAm using 4-cyano-4-(phenyl-carbonothioylthio) pentanoic acid as CTA.
