# Supplementary material for: Temperature- and pH-Responsive Poly(NIPAM-co-HEMA-co-AAm) Nanogel as a Smart Vehicle for Doxorubicin Delivery; Combating Colorectal Cancer
Source: Gels. 2025 Mar 22;11(4):227. doi: 10.3390/gels11040227 (PMC12026571; doi:10.3390/gels11040227)
Supplement: Supplementary file 1 [file gels-11-00227-s001.zip › gels-3357481-supplementary.pdf]

Supporting Information

Temperature- and pH-Responsive Poly(NIPAM-co-HEMA-co-AAm) Nanogel as a Smart Vehicle for Doxorubicin Delivery; Combating Colorectal Cancer

Soheila Ghasemi [1], Mehdi Najafi [1], Mohammad Doroudian [2,*], Banafsheh Rastegari [3], Abbas Behzad-Behbahani [3], Hadis Soltanimehr [1] and Fatemeh Farjadian [4,*]

1 Department of Chemistry, College of Sciences, Shiraz University, 7194684795 Shiraz, Iran
2 Department of Cell and Molecular Sciences, Faculty of Biological Sciences, Kharazmi University, 1571914911Tehran, Iran
3 Diagnostic Laboratory Sciences and Technology Research Center, School of Paramedical Sciences, Shiraz University of Medical Sciences,7143918596 Shiraz, Iran
4 Pharmaceutical Sciences Research Center, School of Pharmacy, Shiraz University of Medical Sciences, 7146864685Shiraz, Iran

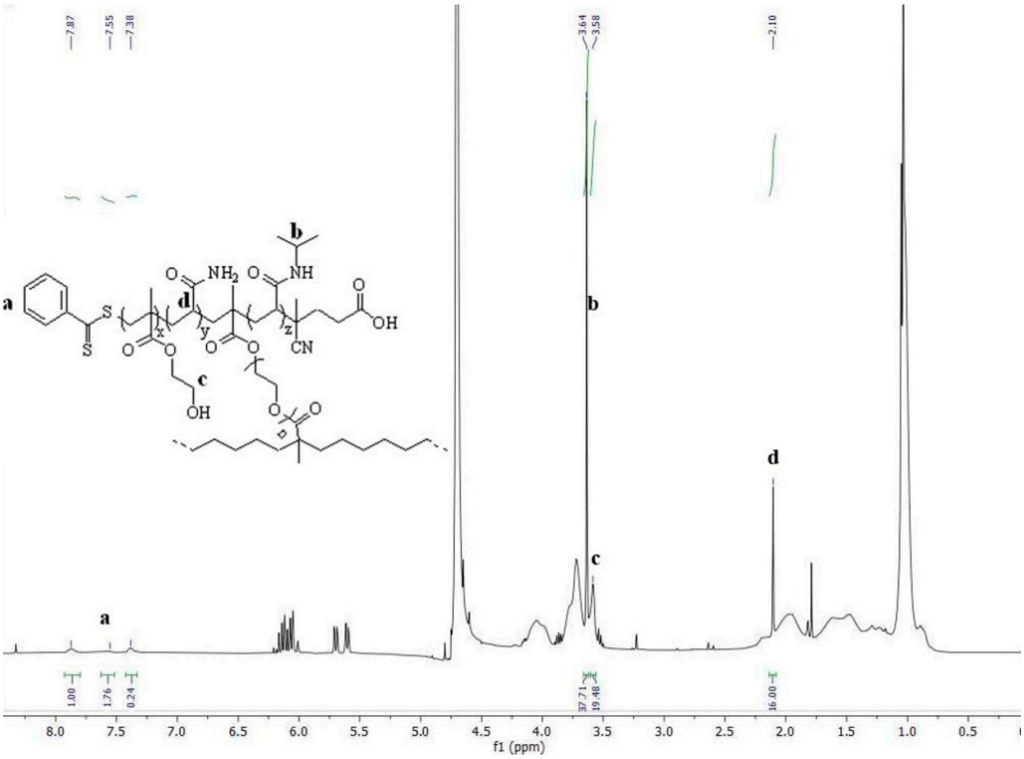

Figure S1: ^{1}H-NMR spectrum of poly (NIPAM-*co*-HEMA-*co*-AAM) hydrogel (**I**) in D_2O

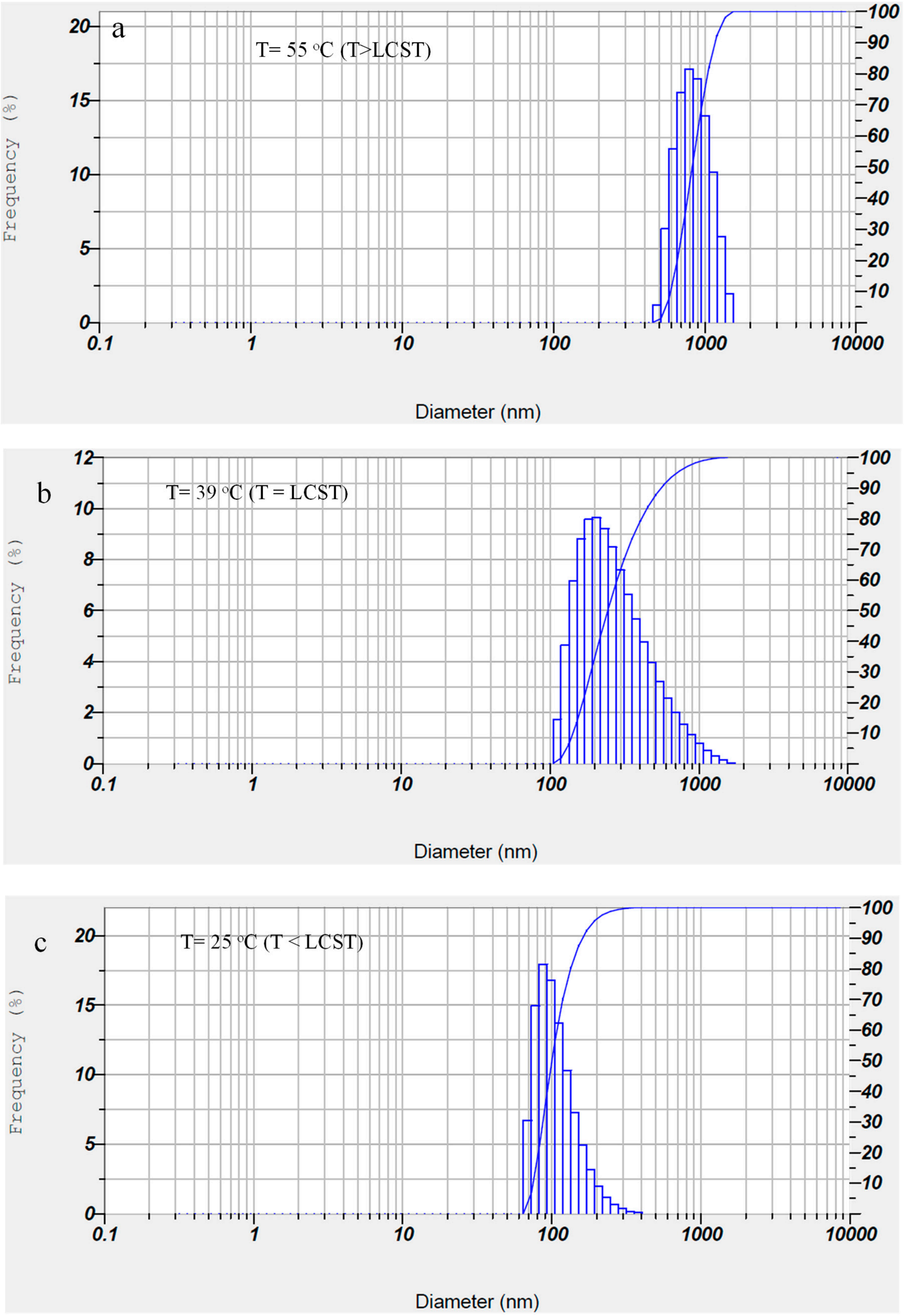

Figure S2: DLS spectra showing the particle size distribution of hydrogel (**I**) at (a) 55 °C (T > LCST), (b) 39 °C (T = LCST), and (c) 25 °C (T < LCST)

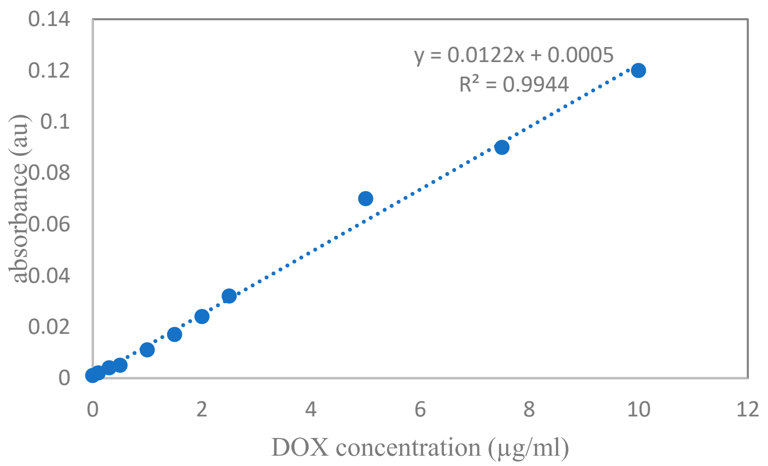

Figure S3: The calibration curve of the DOX

Table S1: RAFT polymerization of NIPAM, HEMA, and AAm using 4-cyano-4-(phenyl-carbonothioylthio) pentanoic acid as CTA

**Hydrogel**	**Feed molar ratio [M]: [CTA]: [In]**	**$DP_{n, NMR}$**	**$DP_{n, th}$**	**$M_{n, NMR}$ [a] [g mol $^{-1}$]**	**$M_{n, th}$ [b] [g mol $^{-1}$]**
Hydrogel (**I**)	100:1:0.2	NIPAM=62.85<br>HEMA=16.23<br>AAm=26.66	NIPAM=60.5<br>HEMA=13.75<br>AAm=25	11811	11104

[a] Calculated by comparing the integration of the peaks of aromatic hydrogens of RAFT agent at 7.38-7.87 ppm with the peaks at 3.64 ppm (NH-**CH**($(CH_3)_2$), 3.58 ppm (CH_2**CH_2**OH) and 2.1 ppm (**CH**$CONH_2$) for NIPAM, HEMA, and AAm repeating units, respectively.

[b] Calculated as, $M_{n,th}$= ([M]/[CTA]) × molecular weight of the monomer and co-monomers unit + molecular weight of the chain ends.
